# *Cuscuta epithymum* Murr. crude extract pre-conditioning protects C6 cells from L-glutamate-induced neurotoxicity

**DOI:** 10.1186/s12906-022-03816-6

**Published:** 2022-12-22

**Authors:** Masoumeh Pourhadi, Zahra Niknam, Rasoul Ghasemi, Mina Soufi Zomorrod, Vahid Niazi, Mehrdad Faizi, Hakimeh Zali, Faraz Mojab

**Affiliations:** 1 Department of Tissue Engineering and Applied Cell Sciences, School of Advanced Technologies in Medicine, Shahid Beheshti University of Medical Sciences, Tehran, Iran; 2 Neurophysiology Research Center, Cellular and Molecular Medicine Institute, Urmia University of Medical Science, Urmia, Iran; 3 Proteomics Research Center, Shahid Beheshti University of Medical Science, Tehran, Iran; 4 Department of Physiology, School of Medicine, Shahid Beheshti University of Medical Sciences, Tehran, Iran; 5 Department of Physiology and Neurophysiology research center, Shahid Beheshti University of Medical Sciences, Tehran, Iran; 6 Department of Hematology and Cell Therapy, Faculty of Medical Sciences, Tarbiat Modares University, Tehran, Iran; 7 Stem Cell Research Center, Golestan University of Medical Science, Gorgan, Iran; 8 Department of Molecular Medicine, Faculty of Advanced Medical Technologies, Gorgan, Iran; 9 Department of Pharmacology and Toxicology, School of Pharmacy, Shahid Beheshti University of Medical Sciences, Tehran, Iran; 10 Medical Nanotechnology and Tissue Engineering Research Center, Shahid Beheshti University of Medical Sciences, Tehran, Iran; 11 Department of Pharmacognosy, School of Pharmacy, Shahid Beheshti University of Medical Sciences, Tehran, Iran

**Keywords:** *Cuscuta epithymum* Murr., Crude extract, Fractions, Phenolic content, Neuroprotective, MDA, SOD, ROS

## Abstract

**Background:**

*Cuscuta epithymum* Murr. *(C. epithymum)*, as an herbal medicine, has played an anti-cancerous role in various studies; however, its possible neuroprotective effects have been neglected. Here, we aimed to investigate the protective effects of *C. epithymum* seeds crude extract and different fractions on rat glioblastoma cells (C6) in L-glutamate oxidative condition.

**Methods:**

Initially, the total phenolic content of *C. epithymum* crude extract and the fractions (all produced by maceration method) was determined. Subsequently, C6 cells were pre-treated with the various concentrations of crude extract and fractions 24 h before L-glutamate exposure. Likewise, C6 cells were treated with the same concentrations of crude extract and fractions 24 h after exposure to L-glutamate. The cell viability and morphology were compared in crude extract and fractions groups, then superoxide dismutase (SODs) activity, reactive oxygen species (ROS), and malondialdehyde (MDA) levels were measured. The flow cytometry test was used to study *C. epithymum* crude extract's effects on the cell cycle and also to quantify the apoptosis, necrosis, and live cells population in different groups.

**Results:**

*C. epithymum* crude extract and fractions (hexanoic, dichloromethanolic, and methanolic) had concentration-dependent cytotoxicity (IC50:126.47, 2101.96, 140.97, and 218.96 µg/ml, respectively). The crude extract and methanolic fraction contained phenolic compounds (55.99 ± 2.795 and 50.80 ± 2.969 mg gallic acid/g extract), while in hexanoic and dichloromethanolic fractions, the phenolic content was undetectable. In the cell viability assay, in comparison to fractions, the crude extract showed a more protective effect against glutamate-induced oxidative condition (*P* < 0.0001). The crude extract increased the SODs activity (*P* < 0.001) and decreased MDA and ROS levels (*P* < 0.0001) in comparison to the glutamate group. The crude extract significantly increased the population of cells in G1 (from 63.04 to 76.29) and decreased the percentage of cells in G2 (from 11.56 to 6.7) and S phase (from 25.4 to 17.01). In addition, it decreased the apoptotic and necrotic cell populations (from 34 to 17.1) and also increased the percentage of live cells (from 66.8 to 83.4 percent) in the flow cytometry test.

**Conclusion:**

*C. epithymum* crude extract plays a neuroprotective role by activating the defense mechanisms in cell against the oxidative condition.

## Introduction

In the central nervous system (CNS), glutamic acid or L-glutamate is a significant excitatory transmitter and a neuronal development mediator [1]. Glutamate receptors are expressed on the surface of brain cells. As a crucial uptake system, glutamate transporters inhibit the over-activation of these receptors by continuously eliminating glutamate from the extracellular fluid in the brain [1]. Excessive activation of glutamate receptors disrupts Ca^2+^ homeostasis and promotes nitric oxide synthesis, free radicals production, and programmed cell death [2, 3]. Besides, excessive extracellular L-glutamate inhibits cystine uptake and blocks glutathione synthesis [4]. Subsequently, the failure of antioxidant defense in cells results in oxidative stress that plays a significant role, along with Ca^2+^ elevated levels, in triggering necrosis and apoptosis in cells. Numerous cellular processes are involved in pathological increasing of extracellular glutamate, including enhanced vesicle exocytosis and also decreased glutamate uptake by excitatory amino acid transporters (EAATs) [5].

Glial cells, as non-neuronal cells, are the most diffused cell type in the CNS and PNS (Peripheral Nervous System) that regulate homeostasis as well as supporting and maintaining neurons [6]. Glial cells express glutamate receptors and have a crucial role in sequestering neuronal-released glutamate through Na^+^-dependent transporters [7]. Recently, several studies have shown that in addition to neurons, excitotoxicity and sustained activation of ionotropic glutamate receptors can lead to glial cell death [8]. It has been proven that some CNS disorders, such as Alzheimer’s disease, Parkinson’s disease, cerebral ischemia, and other diseases, result from excessive excitatory activation of L-glutamate [9].

"Medicinal plants" refers to a diverse range of plants that exhibit therapeutic pharmacological properties. Recently, the modern pharmaceutical industry revolution has led to paying much attention to the development and use of herbal medicines. Based on a World Health Organization (WHO) report, 60% of therapeutic medications are derived from natural sources. In addition, different surveys in various countries have shown that the number of people consuming natural drugs is increasing (WHO Traditional Medicine Strategy 2014–2023) [10]. The Cuscuta genus belonging to the Convolvulaceae plant family has been used in the majority of countries to treat or alleviate a wide variety of disorders, including headache, drooping spirits, epilepsy, nervous and mental disorders, hallucination, amnesia, influenza, fevers, ulcers, and chronic diseases such as diabetes, spleen, liver, joint dysfunctions, spasms, and paralysis for many decades [11]. *Cuscuta epithymum (C. epithymum)* is one of the Cuscuta species named in various countries as Kashout, Pittimo, Aftimoon, crop parasite, among others. *C. epithymum* is a parasitic plant with several secondary metabolites such as glycosides, saponins, tannins, quercetin, steroids, and kaempferol [12]. Recent studies attribute some main biological and pharmacological activities to *C. epithymum*, such as antioxidant, anti-bacterial, anti-fungal, and anti-convulsant activities, urease inhibition, and cytotoxicity [12]. Zhen et al. reported that *Cuscuta chinensis* protects PC12 cells against oxidative stress. The underlying mechanisms may be scavenging reactive oxygen species (ROS) and enhancing the antioxidant enzyme activity [13]. So far, little research has been conducted to show the beneficial, biological, and pharmacological aspects of *C. epithymum* under standard conditions. In this study, we have investigated the protective effects of *C. epithymum* crude extract and fractions in an oxidative in vitro condition.

## Materials and methods

### Extract preparation

#### The methanolic crude extract

*C. epithymum* (Cuscutaceae) seeds were obtained from the Medicinal Plants Store (Tehran, April 2021). F.Mojab taxonomically identified the plant at the School of Pharmacy, Shahid Beheshti University of Medical Sciences, Iran, and registered it with herbarium code SBMU-8236. The extraction process was performed by the maceration method. Briefly, the seeds were grounded and soaked for 24 h in methanol (Merck, Darmstadt, Germany) as a solvent so that it completely covered the grounded seeds. After 24 h of being on the shaker, the top solvent was collected, and the new solvent was poured into the seeds. The procedure was repeated three times and the collected solvent was concentrated in an oven at 40 ℃.

#### The fractions

*C. epithymum* seeds were macerated in n-hexane (Merck, Darmstadt, Germany) for 48 h at room temperature. The extraction procedure was entirely similar to crude extract preparation. After the third collection, the extract was dried at room temperature for 24 h to ensure that all the hexane had been completely removed. The residual plant was macerated further in two other solvents, dichloromethane (Merck, Darmstadt, Germany) and methanol, respectively, in the same manner as n-hexane. By using a rotary evaporator the solvent was removed under a vacuum at 45 ℃. The fractions obtained from each solvent were kept at 4 ℃ for the subsequent study.

### Total phenolic contents measurement

We used the Folin-Ciocalteu analysis method to evaluate the total phenolic content (TPC) in the crude methanolic extract and methanolic, n-hexanoic, and dichloromethanolic fractions. Folin-Ciocalteu solution (Merck, Darmstadt, Germany) was utilized as a reagent, and gallic acid (Merck, Darmstadt, Germany) was the reference phenolic compound. 1 ml of different concentrations of gallic acid, including 0, 10, 20, 40, 60, 80, and 100 g/ml, was combined with 5 ml of diluted Folin-Ciocalteu and incubated at room temperature for 10 min. 4 ml of sodium carbonate solution (75 mg/mL) was added and incubated at room temperature for 30 min. A spectrophotometer was used to determine the absorbance of each standard and sample at 765 nm. For TPC evaluation in samples, 1 ml of the extract with a 400 µg/ml concentration was used. Then, by using the standard curve of gallic acid as a reference, TPC was determined in samples. The data is presented as the average of three independently collected measurements.

### Cell culture

C6 glial cells (rat glioma cell line) were purchased from Pastor Institute (Tehran, Iran) and cultured in 25-cm^2^ culture flasks in DMEM/F12 supplemented with 10% (v/v) fetal bovine serum, 100 U/ml penicillin–streptomycin (Biosera, France) in a humidified atmosphere of 5% CO_2_ at 37 °C. Cells grown to 80–90% confluency were used to perform the following experiments.

#### Determination of L-glutamate cytotoxicity

The cells were sub-cultured in 96-well plates at a density of 8 × 10^3^ cells/well and incubated for 24 h. Adherent cells were exposed to different concentrations of L-glutamate (0.312–20 mM) for 24 h. The morphology of C6 cells treated with the various concentrations of L-glutamate was observed using an inverted light microscope (Olympus, Japan), and the cell viability was evaluated using the 2,5-diphenyl-2H-tetrazolium bromide (MTT) assay (Sigma, Germany). Following 24 h incubation, cells were incubated at 37 °C for an additional 4 h after adding MTT solution at a final concentration of 0.5 mg/ml. 100 μL of DMSO was added to dissolve the formazan crystals after removing the medium. The plate was shaken for 10 min, and the absorbances of the samples were measured at 570 nm by an ELISA reader (BioTek, USA). For all experiments, glutamate was dissolved in DMEM/F12 at final concentrations of 0.312, 0.625, 1.25, 2.5, 5, 10, and 20 mM.

Acridine Orange and Propidium Iodide (AOPI) staining was applied to determine the apoptosis percentage of C6 cells treated with the various concentrations of L-glutamate (Merck, Darmstadt, Germany). For this purpose, 24 h after L-glutamate exposure, the medium was completely removed, and 30 µl of AOPI reagent (Nexcelom Bioscience, Massachusetts, United States) was added to each well. Cells were incubated with AOPI dye for 45 min at 37 °C and observed under a fluorescent microscope (Nikon digital camera, USA).

#### Determination of *C. epithymum* crude extract and fractions cytotoxicity

The cells were sub-cultured in 96-well plates at a density of 8 × 10^3^ cells/well and incubated for 24 h. Adherent cells were exposed to different concentrations (9.37, 19, 37, 75, 150, and 300 µg/ml) of *C. epithymum* crude extract and fractions. The crude extract and fractions were diluted in 100 µL of ethanol (70%) and then in 5 ml of DMEM/F12 to prepare the concentration of 300 µg/ml. The other concentrations were prepared from 300 µg/ml by serial dilution. After 24 h of adding *C. epithymum* crude extract and also fractions into plate, the MTT assay was performed as previously described. The remaining 96-well plates were incubated at 37 °C to assess cell viability after 48 and 72 h. The morphology of cells was observed using an inverted microscope.

#### Effects of *C. epithymum* fractions and crude extract treatment in L-glutamate-induced cytotoxicity

C6 cells were seeded into 96-well plates at 8 × 10^3^ cell density in each well. C6 cells were pre-treated with various concentrations (9.37, 19, 37, 75, and 300 μg/mL) of *C. epithymum* crude extract for 24 h before exposure to L-glutamate. After 24 h, the medium was changed by a fresh culture medium containing L-glutamate (0.312, 2.5, and 10 mM) and incubated for 24 h. In the negative and positive control groups, cells were treated with ethanol (70%) and L-glutamate, respectively. Finally, an MTT assay was performed. Each independent experiment was repeated three times. In parallel to this experiment, post-treatment of C6 cells with *C. epithymum* was performed. In this way, C6 cells were first treated with L-glutamate (0.312, 2.5, and 10 mM) for 24 h. A fresh culture medium containing different concentrations of *C. epithymum* crude extract (9.37, 19, 37, 75, and 300 μg/mL) was added, and the cell viability was evaluated in the same procedure. Three concentrations (19, 37, and 75 μg/mL) were selected for the next experiments to show the protective effects of *C. epithymum* treatment in L-glutamate-induced cytotoxicity.

#### Measurement of superoxide dismutases (SODs) activity and ROS level

SODs are essential enzymes in protecting cells against oxidative damage by reducing superoxide anion free radicals (O2-). SODs activity was measured in cells treated with L-glutamate and *C. epithymum* crude extract. C6 cells were grown in 25 cm ^2^ flasks for pre-treatment and post-treatment experiments with 19, 37, and 75 g/ml crude extract and 10 mM glutamate to measure SODs activity. By using a rubber policeman cells were removed from the flasks 24 h after the last treatment. Two million cells were required for the test. The cell pellet was sonicated for 30 s at medium power (10 s of sonication plus 10 s of rest; 3 times) before being centrifuged at 12,000 g for 5 min at 4° C. The supernatant was separated and placed on ice. According to the kit protocol (Teb Pazhouhan Razi (TPR) Innovative, Tehran, Iran), samples and working solution (chromogenic reagent and SODS assay buffer) were added to 96-well plates. Eventually, the SODS enzyme solution was added and incubated for 20 min. After 20 min, a multi-mode reader read the absorbance at a 440–460 nm wavelength (BioTek, USA). ROS level was also measured by kit (Teb Pazhouhan Razi (TPR) Innovative, Tehran, Iran) via 2′-7′-dichlorodihydrofluorescein diacetate assay (DCFH-DA). For this experiment, C6 cells were seeded into 96-well plates at 8 × 10^3^ cells density in each well and treated with crude extract similar to whatever was conducted for the SOD test, and 24 h after the last treatment the cells were incubated with DCFH-DA. After 30 min the fluorescence was measured at 510–550 nm wavelength by a microplate reader (BioTek, USA).

#### Malondialdehyde (MDA) assay

Malondialdehyde (MDA) is an oxidative stress indicator and the most common reactive aldehyde produced during lipid peroxidation. In this study, the thiobarbituric acid reactive substances (TBARS) were measured to determine the products of lipid peroxidation. After 24 h of the last treatment, the cells were trypsinized for the MDA test. According to the kit (TPR Innovative, Iran), one million cells were required in 1 ml of PBS buffer (pH: 7.8). First, 10 µL of butylated hydroxytoluene (BHT) was added to the cells. Cells were then placed on ice and sonicated with a medium-power sonicator for 30 s. Detergent was added to all samples and incubated at 37 ˚C. Then, the chromogenic solution containing thiobarbituric acid, acetic acid, and alkali was added to the samples and placed in boiling water for an hour. After one hour, they were placed on ice for 10 min, then centrifuged at 10,000 g for 10 min at 4 °C. The supernatant was transferred to the 96-cell plate, and the absorbance of the groups was measured using a multi-mode reader at a wavelength of 530–540 nm. Protein concentrations were measured in samples by the multi-mode reader at 280 nm.

#### Effects of *C. epithymum* crude extract on the apoptosis of C6 cells in L-glutamate-induced damage condition

At the end of the final 24 h of cells incubation with *C. epithymum* crude extract and L-glutamate, C6 pre-treated and post-treated cells were harvested and centrifuged at 1500 rpm for 5 min. For determining the percentage of the apoptotic cells, Annexin V-FITC (fluorescein isothiocyanate)/PI (propidium iodide) (Biolegend, USA) co-staining method was used. For this purpose, 500 µL of 1X binding buffer was added to the cell pellet, and the sample was divided into tubes. 5 µL of Annexin V-FITC and 3 µL of PI were added to labeled tubes and then incubated at 4 °C for 15 min in the dark. Then, the specific fluorescence of cells was detected using a BD FACS Calibur (BD Biosciences, San Jose, CA, USA) and the outcomes were evaluated by FlowJo software.

#### Effects of *C. epithymum* crude extract on the C6 cell cycle in L-glutamate-induced damage condition

At the end of the final 24 h of cells incubation with *C. epithymum* crude extract and L-glutamate, C6 pre-treated and post-treated cells were harvested and centrifuged at 1500 rpm for 5 min. Then 50 µL of cold PBS was added to the cells and gently vortexed. The cells were resuspended, fixed with cold 70% ethanol, and vortexed gently. After fixation by ethanol, the cells were washed with PBS and centrifuged at 1500 rpm for 5 min. The pellet was resuspended in 1 ml of PI master mix containing 40 µL of PI (1 mg/ml), 950 µL of PBS, and 10 µl of RNase (DNase free, 10 mg/ml) and incubated for a half-hour at room temperature. Finally, the cells were analyzed by flow cytometry (BD FACSCalibur, BD Biosciences, USA), and the outcomes were evaluated by FlowJo software.

### Statistical analysis

Each experiment was conducted at least three times, and the data were presented as the mean ± standard deviation (SD). Statistical analysis for cell viability tests, SOD, and MDA were performed using one-way ANOVA and appropriate posthoc tests in GraphPad Prism 8.4.3. Two-way ANOVA and appropriate posthoc test analyzed the apoptosis and cell cycle data. The *P* < 0.05 indicated that the result was statistically significant.

## Results

### Yield extract and total-phenolic content

The crude and fractions extraction yield and TPC results are presented in Table 1. The yield of the crude extract was about 18.26%, and n-hexanoic, dichloromethanolic, and methanolic fractions were approximately 0.89%, 1.33%, and 28.59%, respectively. This experiment showed that the *C. epithymum* crude methanolic extract contains more phenolic compounds, followed by methanol fraction in comparison with other fractions.

**Table 1:** The extraction yield and total phenolic content of the extract and the fractions

	**Fractions**			
	Crude extract	n-hexane	Dichloromethane	methanol
**Yield extract (%)**	18.26	0.89	1.33	28.59
**TPC (mg gallic acid/g extract)**	55.99 ± 2.795	Undetectable	Undetectable	50.80 ± 2.969

### Cytotoxicity of L-glutamate in a concentration-dependent manner

**Fig. 1:**
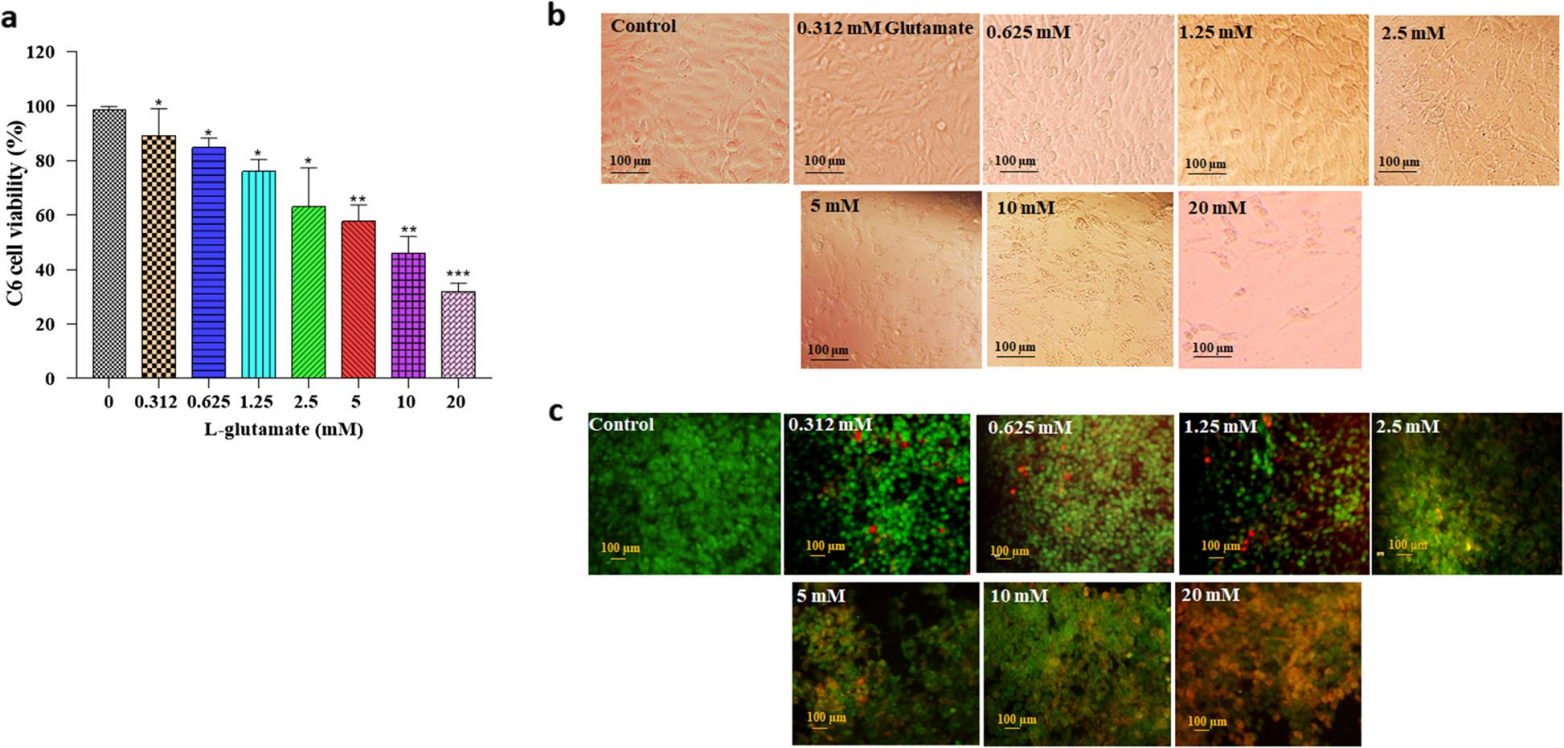
Effects of L-glutamate on C6 cell viability. **a** MTT assay showed that cell survival is dependent on glutamate concentration. AO/PI results were compatible with the MTT assay. Increased L-glutamate concentration led to significantly decreased cell viability. The data are expressed as mean ± standard deviation. *P < 0.05, **P < 0.01, ***P < 0.001. **b** Observation of cells under the inverted microscope with a 20X c) AOPI fluorescence images of C6 cells in the presence of different glutamate concentrations

In this study, the MTT assay was used to determine the viability of C6 cells in various L-glutamate concentrations exposure (0, 0.312, 0.625, 1.25, 2.5, 5, 10, and 20 mM). Results demonstrated that increased L-glutamate concentration significantly decreased the percentage of viable cells. Comparing 10 mM glutamate to the control group showed that cell viability decreased by approximately 50 percent (*P* < 0.01) (Fig. 1a). Moreover, the inverted and fluorescent microscope images showed apoptotic morphological and color changes in the L-glutamate-treated cells (Fig. 1b, c). The existence of apoptotic cells in the treated cells was indicated by the presence of a red and bright orange stain, in contrast to the bright green coloring of the living cells. By increasing L-glutamate concentration, the proportion of green cells gradually reduced while the percentage of orange cells enhanced, indicating a transition from viable to apoptotic cells. In the current study, a 10 mM concentration of L-glutamate was chosen for the subsequent experiments.

### Cytotoxicity of *C. epithymum* crude extract and fractions

**Fig. 2:**
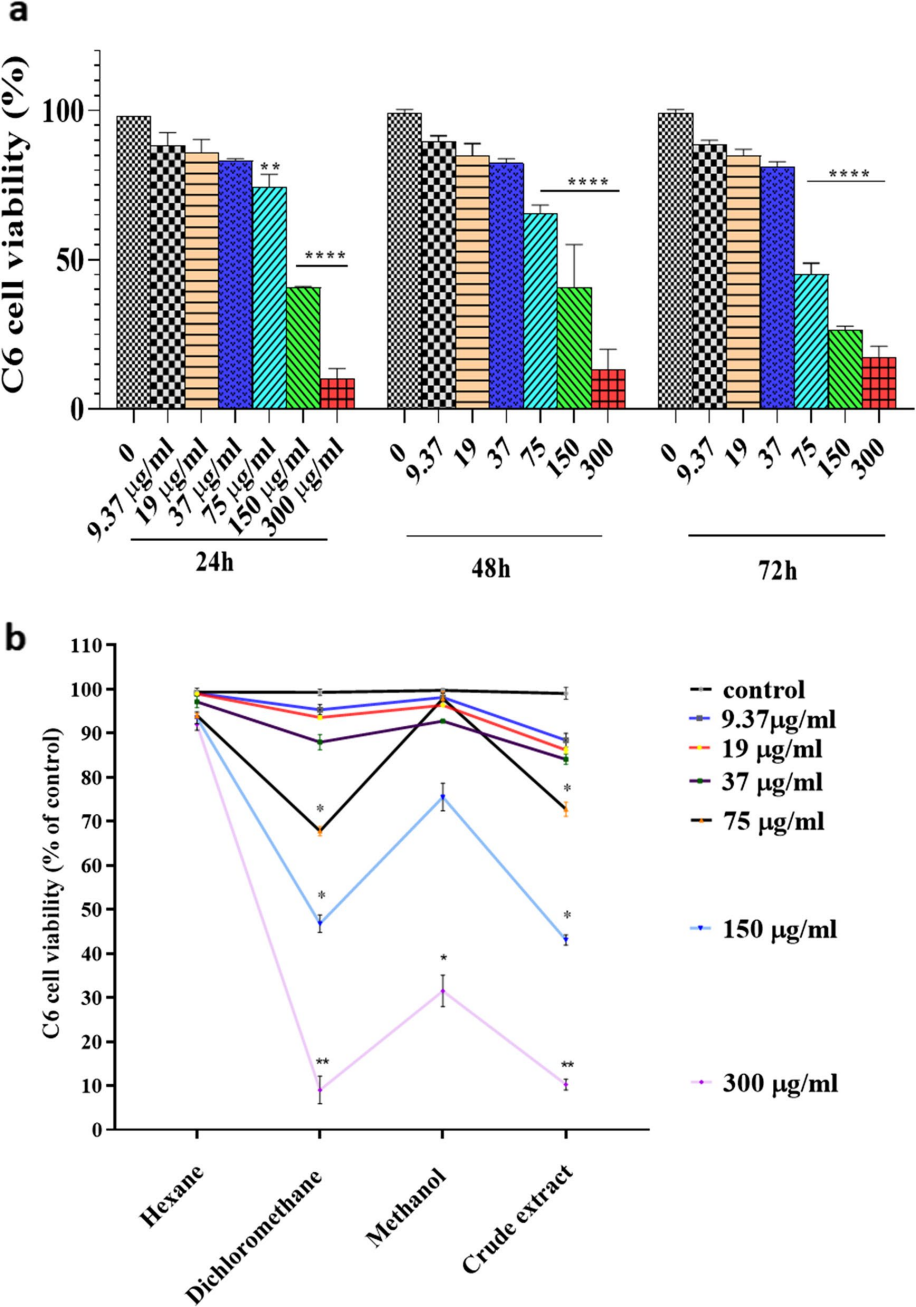
Effects of *C. epithymum* extract on cell survival in C6 cells. **a** MTT assay of crude extract. **b** Viability of cells in the presence of crude extract and fractions (average of three days results). At the high concentrations of *C. epithymum crude* extract, dichloromethanolic, and methanolic fractions, the cell viability significantly decreased. The data are expressed as mean ± standard deviation. *P < 0.05, **P < 0.01, ***P < 0.001, ****P < 0.0001

At different concentrations (9.37, 19, 37, 75, 150, and 300 µg/ml), the cytotoxicity of *C. epithymum* methanolic crude extract and the n-hexanoic, dichloromethanolic, and methanolic fractions were studied to choose the optimum concentration for cell treating at the subsequent steps. Results of the MTT assay for the crude extract after 24, 48, and 72 h incubation showed that *C. epithymum* crude extract at high concentrations decreased the cell viability (Fig. 2a). It means high concentrations (75, 150, and 300 µg/ml) have a significant toxic effect on C6 cells and lead to cell death (*P* < 0.0001). The concentrations lower than 75 µg/ml did not show significant toxic effects.

Moreover, comparing the toxicity of *C. epithymum* crude extract and the fractions indicated that the 9.37, 19, and 37 µg/ml concentrations did not show a significant toxic effect on the cells (Fig. 2b). Dichloromethanolic fraction at high concentrations (75, 150, and 300 µg/ml) showed significant toxic effects similar to the crude extract. In contrast, the methanolic fraction at high concentrations indicated lower toxicity compared with the dichloromethanolic fraction and the crude extract. The n-hexanoic fraction did not show cytotoxicity at any concentration. The concentration of crude extract, hexanoic, dichloromethanolic, and methanolic fractions extract that induced 50 percent cell death in C6 cells were 126.47, 2101.96, 140.97, and 218.96 µg/ml, respectively.

### Effects of C. epithymum crude extract and fractions on C6 cell viability in L-glutamate-induced condition

**Fig. 3:**
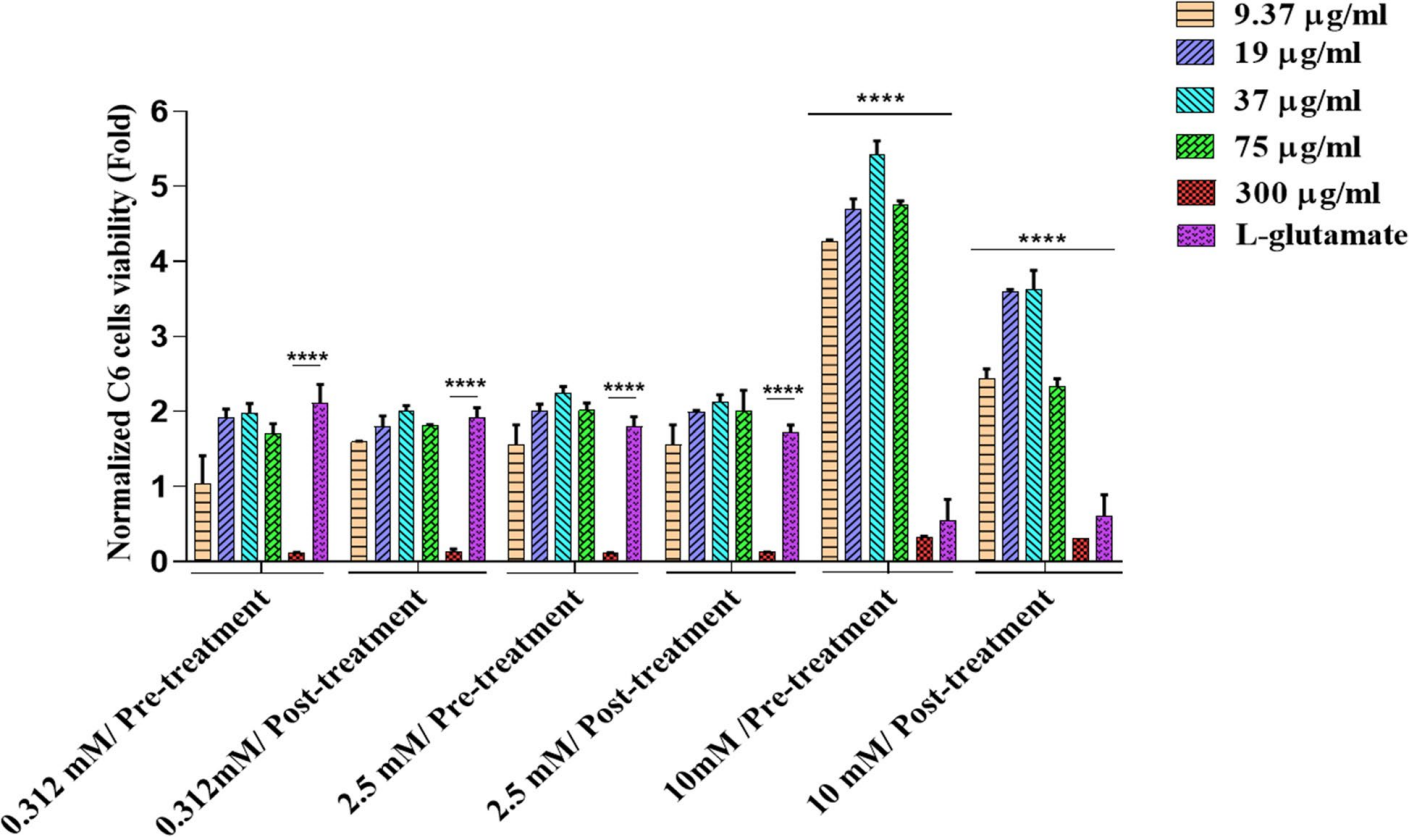
Viability of cells in pre-treatment and post-treatment with crude extract, in the presence of L-glutamate using MTT assay. Pre-treatment and post-treatment of the crude extract in the presence of a 10 mM concentration of L-glutamate caused a significant increase in cell viability compared to the L-glutamate group. The data are expressed as mean ± standard deviation. ****P < 0.0001

This investigation aims to determine whether *C. epithymum* extract protects C6 cells against L-glutamate-induced damage or not. We chose 9.37, 19, and 37 µg/ml as optimum concentrations, and 75 and 300 µg/ml as high concentrations of the crude extract. In pre-treatment groups, the crude extract was added to the cells for 24 h before removing the medium and adding the fresh medium containing glutamate (0.312, 2.5, and 10 mM). In post-treatment groups, the cells were exposed to glutamate, and after 24 h, a fresh medium containing the crude extract was added to the culture medium. Then, after 24 h of incubation, an MTT assay was performed. Results showed that 0.312 and 2.5 mM L-glutamate are not suitable concentrations for the study. There were no significant changes between *C. epithymum* crude extract and L-glutamate treated groups in these concentrations in cell viability (Fig. 3). But compared with 10 mM concentration L-glutamate, crude extract pre-treatment and post-treatment significantly changed cell viability (*P* < 0.0001). The crude extract with 9.37, 19, 37, and 75 µg/ml concentrations significantly protected the survival of cells in L-glutamate high doses exposure. So we chose a 10 mM concentration of L-glutamate to demonstrate *C. epithymum* fractions' protective effects on C6 cells.

**Fig. 4:**
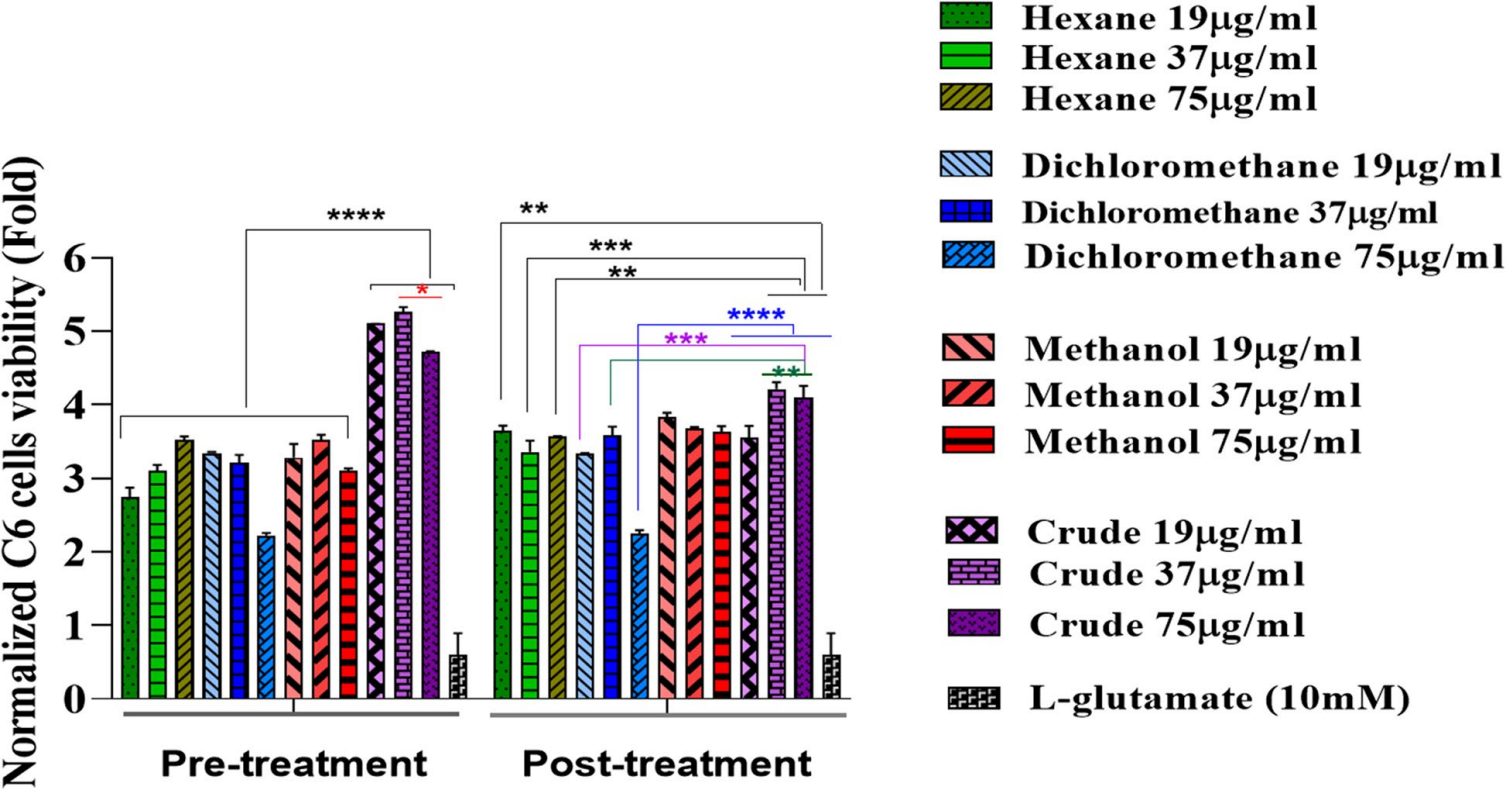
Viability of cells in pre-treatment and post-treatment with crude extract and fractions, in the presence of 10 mM L-glutamate using MTT assay. The crude extract and fraction significantly enhanced cell viability in L-glutamate-treated groups. Crude extract showed more effectiveness on cell viability. The data are expressed as mean ± standard deviation. *P < 0.05, **P < 0.01, ***P < 0.001, ****P < 0.0001

In the next step, we comprised the impact of concentrations (19, 37, and 75 µg/ml) of *C.epithymum* crude extract and three fractions on cell viability in the presence of 10 mM of L-glutamate (Fig. 4). Cell viability in the L-glutamate pre-treatment and the post-treatment groups, without the intervention of extracts and fractions, was significantly decreased. However, the crude extract and the fractions significantly enhanced cell viability in L-glutamate-treated groups compared to the glutamate-exposed C6 cells without extract treatment. In addition, the data suggested that the viability of cells in the pre-treatment and the post-treatment with crude extract are significantly more than the fractions-treated groups, and crude extract of *C. epithymum* inhibits the toxic effects of L-glutamate more efficiently.

### Effect of C. epithymum crude extract on oxidative stress markers and ROS production in cytotoxic condition

**Fig. 5:**
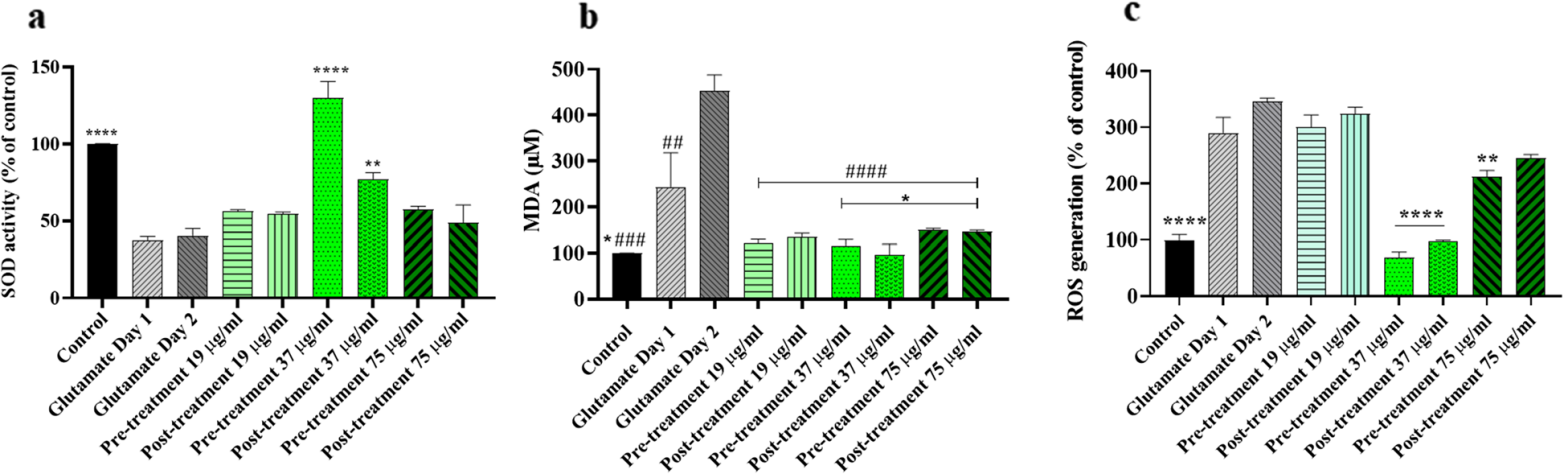
Effect of *C. epithymum* crude extract on levels of oxidative stress factors in C6 Cells after L-glutamate-induced cytotoxicity. **a** Superoxide dismutase (SODs) activity. Pre-treatment with 37 µg/ml *C. epithymum* crude extract significantly increased SODs activation against L-glutamate-induced oxidative stress. The data have been compared with glutamate day 1 and day 2 **b** Malondialdehyde (MDA) contents. All concentrations of the crude extract significantly decreased the MDA level. * Compared to glutamate day 1 # compared to glutamate day 2. **c** ROS generation in different groups. The data were compared with glutamate on day 1 and day 2. The data are expressed as mean ± standard deviation. *P < 0.05, **P < 0.01, ***P < 0.001, ****P < 0.0001

As shown in Fig. 5, SOD, MDA, and ROS levels were measured in the *C. epithymum* crude extract-treated cells in the presence of L-glutamate as an oxidative stress inducer. SODs levels, as an antioxidant enzyme, in the L-glutamate treated groups without extract intervention were significantly decreased compared with the control group (*P* < 0.01). The evidence proved that pre-treatment with 37 µg/ml *C. epithymum* crude extract significantly increased SODs activation against L-glutamate-induced oxidative stress (*P* < 0.001) (Fig. 5a). SODs levels were not significantly changed in the other concentrations of the extract. As shown in Fig. 5b, the MDA level was significantly enhanced in the L-glutamate groups, especially on the second day group (glutamate was added to culture medium as a post-treatment agent on the second day of study), compared to the control group (*P* < 0.0001). All concentrations of the extract (19, 37, and 75 µg/ml) significantly decreased the MDA level compared to the L-glutamate-non-extract-treated groups (*P* < 0.0001). To study whether the crude extract may involve in decreasing intracellular ROS production, we measured the ROS level in C6 cells after treatment with the different concentrations of crude extract. According to Fig. 5c, the pre-treatment and post-treatment of cells with a 37 µg/ml concentration of crude extract significantly decreased ROS levels in cells. The pre-treatment with a 75 µg/ml concentration decreased the ROS level, as well. Interestingly, in the group of 19 µg/ml concentration, there were no significant changes in comparison to the glutamate group.

### Effects of C. epithymum crude extract on the cell cycle and apoptosis in cytotoxic condition

**Fig. 6:**
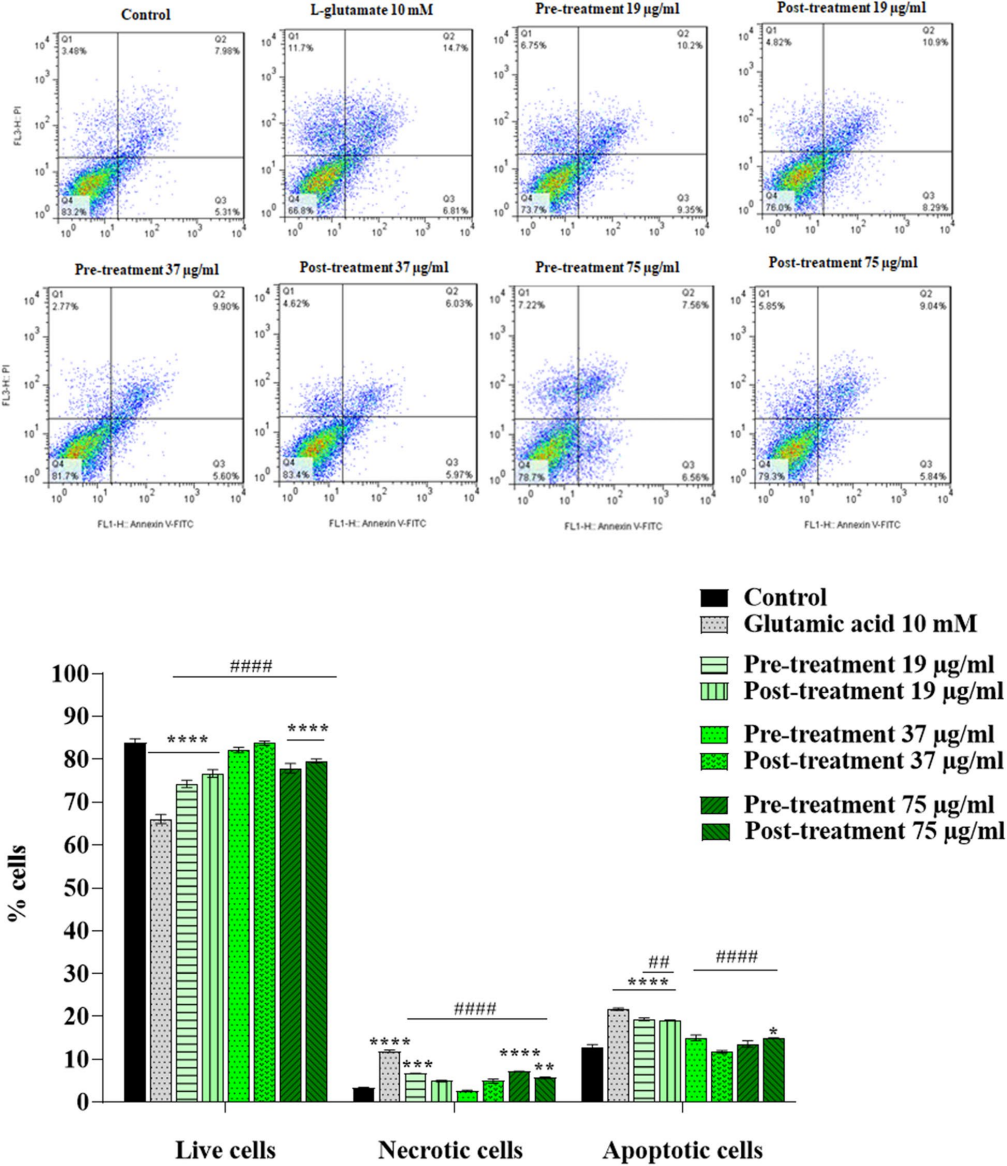
Effect of *C. epithymum* crude extract on C6 cells apoptosis in cytotoxic condition induced by L-glutamate. Pre-treatment and post-treatment with crude extract significantly decreased the percentage of apoptotic cells. The data are expressed as mean ± standard deviation. *P < 0.05, **P < 0.01, ***P < 0.001, ****P < 0.0001 as compared to control-untreated group; ##*P* < 0.01, ####*P* < 0.0001 as compared to L-glutamate-treated group

To determine the proportion of apoptotic cells in pre-treated and post-treated cells with different concentrations (19, 37, and 75 µg/ml) of *C. epithymum* crude extract and L-glutamate (10 mM), we used Annexin V and PI co-staining assay. In this method, flow cytometry analysis plots indicate four regions: 1) viable cells (negative for Annexin V and PI), 2) early apoptotic cells (positive for Annexin V but negative for PI), 3) late apoptotic cells (positive for Annexin V and PI), and 4) necrotic cells (negative for Annexin V but positive for PI) [10]. As shown in Fig. 6, all the groups, including the control and the treated cells with L-glutamate and *C. epithymum* crude extracts, indicated the apoptotic and necrotic cell percentage after 24 h of incubation. Pre-treatment and post-treatment with 19 µg/ml (*P* < 0.01), 37, and 75 µg/ml (*P* < 0.0001) of the crude extract significantly reduced the percentage of the apoptotic cells in comparison with the L-glutamate treated cells. Pre-treatment and post-treatment with all concentrations (19, 37, and 75 µg/ml) of crude extract in the presence of L-glutamate not only significantly decreased the percentage of necrotic cells but also increased the percentage of the live cells in comparison with the L-glutamate-treated groups without extract (*P* < 0.0001).

**Fig. 7:**
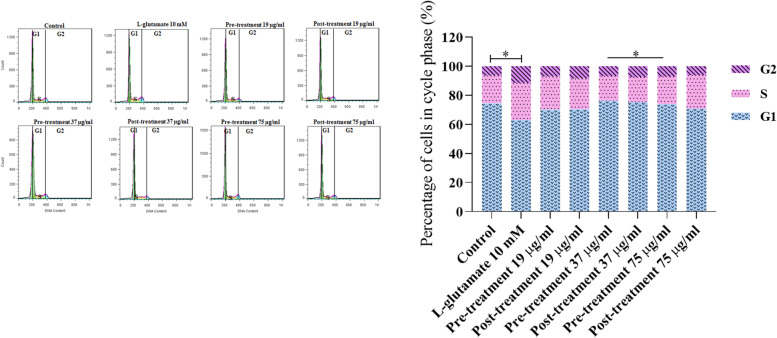
Effect of *C. epithymum crude extract* on cell cycle in C6 cells after L-glutamate-induced cytotoxicity. Pre-treatment and post-treatment with crude extract at 37 and 75 g/ml concentrations had a positive effect on the cell cycle and significantly increased the population of cells in G1, and decreased the percentage of G2 and S phase cell population. The data are expressed as mean ± standard deviation. *P < 0.05 as compared to the L-glutamate-treated group

In to cell cycle analysis in the pre-treated and post-treated cells, DNA content detection was conducted using the Propidium Iodide staining method. The results were presented in percentages in Fig. 7. We found a reduction in S and G2 phases and an enhancement in the G1 phase in C6 cells at 37 and 75 g/ml concentrations, indicating that the crude extract inhibits S arrest in C6 cells.

## Discussion

This study evaluated the protective effect of *C. epithymum* crude extract and fractions on C6 cells in oxidative stress conditions induced by L-glutamate. The results showed that *C. epithymum* methanolic crude extract could efficiently decrease the cytotoxicity effects of L-glutamate. Pre-treatment and post-treatment of the cells with low concentrations of *C. epithymum* extracts decreased dead cell percentage in the presence of L-glutamate-induced cytotoxicity and increased cell viability. In addition, *C. epithymum* crude extract reduced MDA and ROS, while enhancing SODs levels in the C6 cells.

Glutamate, an abundant excitatory neurotransmitter in mammalians' central nervous system, plays a crucial role in fast synaptic transmission, neuronal development, pain perception, learning, and memory [14]. Because of increased release or/and uptake disorders, the excess glutamate level has been demonstrated to stimulate significant neurotoxicity and neuronal death in brain tissue [15]. The neurotoxicity can be related to the enhanced damage in cell components, such as mitochondria and membrane depolarization, causing cell death and the production of reactive oxygen species (e.g., superoxide anion (O_2_^−^**•**), hydroxyl radical (OH•), and hydrogen peroxide (H_2_O_2_)) [16, 17]. The stimulation of oxidative stress by glutamate has been indicated in cell lines, including C6, PC-12, oligodendroglia cells, and immature cortical neurons [17].

Since glutamate receptor subtypes (ionotropic and metabotropic) are expressed in C6 glioblastoma cells, these cells can be a beneficial model for investigating excitotoxicity [18]. Moreover, C6 glial cells provide physical and metabolic support to the survival of neurons, such as insulation and communication of neurons and transportation of nutrients and waste; hence, glial cell damage may play role in the neurodegeneration process. Recent investigations show that astroglial cell damage is strongly related to many neurological disorders, including Parkinson’s disease, Alzheimer’s disease, and ischemic stroke [18]. In the in vitro toxicity studies, depending on the culture conditions, the L-glutamate concentration ranges from 0.01 to 20 mM [19]. We selected 10 mM L-glutamate in our study since, at this concentration, approximately 50% cell viability was detected and the effect of *C. epithymum* on neutralizing the cytotoxicity of L-glutamate was significant.

*C. epithymum* is a widely used parasitic plant in agriculture and food industries, pharmaceutical, cosmetics, and sanitary due to its fungicidal, virucidal, bactericidal, anti-parasitical, and insecticidal properties [20]. Recent findings have also shown that *C. epithymum* has neuropharmacological and sedative-hypnotic effects without significant toxic influence in mice. This plant's sedative-hypnotic properties are mediated through benzodiazepine receptors [21]. Due to its antioxidant properties, we selected this medicinal plant to evaluate its effect on L-glutamate-induced damage in C6 cells. Ganapathy et al. also reported antioxidant and free radical scavenging properties of *C. epithymum* depend on the doses [22].

This study investigated the extraction yield and TPC of *C. epithymum* crude extract and the fractions. Different chemicals can be obtained in the extraction procedure due to the different solvent polarity, the various chemical composition of the plant, and the presence of interfering materials [23]. Methanol showed the most yield percentage in the extraction procedure, followed by dichloromethane and n-hexane. Therefore, we can infer that the solvent with more polarity will produce a higher extraction yield. The TPC results showed that the crude methanol extract has the highest total phenolic content, suggesting that the solvent polarity influences the extraction of various phenolic chemicals from *C. epithymum*. We showed that C6 cells treated with different concentrations of L-glutamate (24 h) resulted in cell damage and cell viability decrease. According to the results, we suggested that low concentrations of *C. epithymum* crude extract and the fractions can enhance cell survival and counteract the damaging effects of L-glutamate. Considerably, results demonstrated that the crude extract compared with the fractions had more impact on the protection and survival of cells. We used the crude extract for further tests, including antioxidant, cell cycle, and apoptosis assays. We also showed that *C. epithymum* crude extract and fractions at high concentrations have cytotoxic effects and reduce cell viability. Our findings were consistent with the findings of earlier studies indicating that a high concentration of *C. epithymum* extracts shows cytotoxicity and can be used for inducing apoptosis in cancer cells [24, 25].

Under oxidative stress conditions, the organism's oxidant and antioxidant defense mechanisms become unbalanced, leading to excessive ROS production, tissue damage, and physiological function impairment [26]. In addition, oxidative stress is one of the most critical risk factors for neurodegenerative conditions and CNS disorders [27]. According to previous studies, *C. epithymum* showed dose-dependent scavenging activity using DPPH, superoxide, and hydroxyl radical scavenging assays [28]. Qualitative phytochemical studies on the *C. epithymum* also manifested the presence of alkaloids, triterpenoids, flavonoids, steroids, glycosides, and carbohydrates, which might be responsible for its antioxidant properties [28]. Another electrochemical assay study demonstrated that *C. epithymum* has desirable antioxidant properties [20]. Consistent with these studies, in our study, the antioxidant assays (SODs, ROS, and MDA) revealed that the relative antioxidant ability of *C. epithymum* crude extract plays a scavenger role in C6 cells against free radicals produced in the presence of L-glutamate.

Long-term glutamate accumulation in the intercellular space stimulates neurotoxic effects. It elevates Ca levels in cells- an imbalance of electrogenic ions gradients- and triggers various intracellular cascades that lead to plasma membrane damage and neuron apoptosis [29]. In our study, L-glutamate stimulates apoptosis and necroptosis in C6 cells based on flow cytometry analyses. Pre- and post-treatment with *C. epithymum* crude extract prevented apoptosis and necroptosis induced by L-glutamate. A study in line with our results indicated that glutamate stimulates apoptosis and necroptosis in C6 cells [18].

## Conclusion

Our research indicates that *C. epithymum* hexanoic fraction has no cytotoxic effect on C6 cells. Although it has an undetectable amount of phenolic content, it can slightly protect C6 cells against the oxidative condition. Among different fractions of *C. epithymum,* dichloromethanolic fraction has a more cytotoxic effect than methanolic and hexanoic fractions. It has an undetectable amount of phenolic content and can slightly protect C6 cells against glutamate cytotoxic effects. The methanolic fraction has a considerable amount of phenolic content and less cytotoxic effect than methanolic crude extract. Nevertheless, the methanolic fraction is not as protective as a methanolic crude extract in exposure to an oxidative environment. Although the methanolic crude extract has a significant amount of phenolic compounds, it has a considerable cytotoxic effect at high doses. The crude extract provides more cell protection than different fractions and this observation may be mediated by the activation of defense mechanisms of cells against reactive oxygen species (ROS) and also the antioxidant activities of extract. For instance, the delay in the cell cycle, producing enzymes, including catalase, peroxidase, and superoxide dismutase along with antioxidant synthesis are defensive responses against ROS in cells. It seems that crude extract not only decreases membrane phospholipids peroxidation and ROS production but also induces enzyme production like SOD and changes the cell cycle. The phenolic compound in the crude extract may have a direct effect on radical scavenging and decreasing the ROS and MDA levels in treated cells. The decreased dead cell population and increased G1 cell population can be attributed to the phenolic content and the antioxidative role of extract. The crude extract has the highest antioxidant activity to significantly increase C6 cell viability in cytotoxic conditions. It probably plays a significant role in reducing cell death by decreasing MDA levels and increasing SODs levels in cells, showing lipid peroxidation prevention and free radical scavenging, respectively. All of these attributes result in decreasing cell death by treating cells with the crude extract. Nonetheless, further research is required to evaluate the active component(s) and molecular mechanisms (apoptosis and necrosis) involved in *C. epithymum* neuroprotective effects. The apoptotic pathways and involved pre-apoptotic and anti-apoptotic molecules can be studied in the future. Furthermore, the oxidative stress gene expression is another aspect of the future study for assessing the neuroprotective effects of the crude extract.

## Data Availability

Most of the data are included in this published article and further data are available from the corresponding author on request.
